# No improvement in the reporting of clinical trial subgroup effects in high-impact general medical journals

**DOI:** 10.1186/s13063-016-1447-5

**Published:** 2016-07-16

**Authors:** Nicole B. Gabler, Naihua Duan, Eli Raneses, Leah Suttner, Michael Ciarametaro, Elizabeth Cooney, Robert W. Dubois, Scott D. Halpern, Richard L. Kravitz

**Affiliations:** Center for Clinical Epidemiology and Biostatistics, Perelman School of Medicine, University of Pennsylvania, 423 Guardian Drive, 708 Blockley Hall, Philadelphia, PA 19104 USA; Department of Psychiatry and New York Psychiatric Institute, Columbia University, New York, NY USA; Department of Biostatistics, Perelman School of Medicine, University of Pennsylvania, Philadelphia, PA USA; National Pharmaceutical Council, Washington, DC, USA; Department of Medicine, Pulmonary, Allergy, and Critical Care Division, Perelman School of Medicine, University of Pennsylvania, Philadelphia, PA USA; Department of Internal Medicine, Division of General Medicine, University of California Davis School of Medicine, Sacramento, CA USA

**Keywords:** Randomized controlled trial, Heterogeneity of treatment effects, Subgroup analysis, Methodology, Multivariable risk index

## Abstract

**Background:**

When subgroup analyses are not correctly analyzed and reported, incorrect conclusions may be drawn, and inappropriate treatments provided. Despite the increased recognition of the importance of subgroup analysis, little information exists regarding the prevalence, appropriateness, and study characteristics that influence subgroup analysis. The objective of this study is to determine (1) if the use of subgroup analyses and multivariable risk indices has increased, (2) whether statistical methodology has improved over time, and (3) which study characteristics predict subgroup analysis.

**Methods:**

We randomly selected randomized controlled trials (RCTs) from five high-impact general medical journals during three time periods. Data from these articles were abstracted in duplicate using standard forms and a standard protocol. Subgroup analysis was defined as reporting any subgroup effect. Appropriate methods for subgroup analysis included a formal test for heterogeneity or interaction across treatment-by-covariate groups. We used logistic regression to determine the variables significantly associated with any subgroup analysis or, among RCTs reporting subgroup analyses, using appropriate methodology.

**Results:**

The final sample of 416 articles reported 437 RCTs, of which 270 (62 %) reported subgroup analysis. Among these, 185 (69 %) used appropriate methods to conduct such analyses. Subgroup analysis was reported in 62, 55, and 67 % of the articles from 2007, 2010, and 2013, respectively. The percentage using appropriate methods decreased over the three time points from 77 % in 2007 to 63 % in 2013 (*p* < 0.05). Significant predictors of reporting subgroup analysis included industry funding (OR 1.94 (95 % CI 1.17, 3.21)), sample size (OR 1.98 per quintile (1.64, 2.40), and a significant primary outcome (OR 0.55 (0.33, 0.92)). The use of appropriate methods to conduct subgroup analysis decreased by year (OR 0.88 (0.76, 1.00)) and was less common with industry funding (OR 0.35 (0.18, 0.70)). Only 33 (18 %) of the RCTs examined subgroup effects using a multivariable risk index.

**Conclusions:**

While we found no significant increase in the reporting of subgroup analysis over time, our results show a significant decrease in the reporting of subgroup analyses using appropriate methods during recent years. Industry-sponsored trials may more commonly report subgroup analyses, but without utilizing appropriate methods. Suboptimal reporting of subgroup effects may impact optimal physician-patient decision-making.

**Electronic supplementary material:**

The online version of this article (doi:10.1186/s13063-016-1447-5) contains supplementary material, which is available to authorized users.

## Background

Heterogeneity of treatment effects (HTE) exists when not all patients respond to a treatment in a similar fashion [1]. Understanding HTE will allow providers to target treatments and provide the best guidance to patients who are most likely to benefit. Multiple examples of clinically important HTE have been shown in the literature [2–5], and more efficient targeting of treatment is not only a better use of resources, but can also reduce side effects and other adverse outcomes. However, the only way to determine which groups of patients are most likely to incur a net benefit is to examine treatment effects across subgroups of patients [6, 7].

Statistical methods for examining HTE across subgroups of patients (“subgroup analysis”) have been well described [6–8]. These methods are included in guideline documents such as the CONSORT statement [9], as well as in documented methodology standards for the Patient-Centered Outcomes Research Institute (PCORI) [10]. While studies have examined subgroup reporting and methodology in general medical journals [11–16], subspecialties [17, 18], and surgery [19], understanding the underlying factors that influence reporting and use of appropriate methodology has not been well explored. Indeed, the most comprehensive study to date [13] was limited to 1 year (2007) and did not differentiate between appropriate and inappropriate methodology for subgroup analysis. An earlier review [16] that examined subgroup reporting during the years 1994, 1999, and 2004 found that subgroup analysis was reported in less than 60 % of randomized controlled trials (RCTs) and correctly analyzed approximately half the time, although this study did not explore the predictors of appropriate statistical methodology. Furthermore, little research exists regarding which variables are selected for subgroup analysis. Single variables are often the most simple to examine but can only assess a single dimension of risk. Recent simulations suggest it may be far preferable to assess treatment effect heterogeneity across groups defined by simultaneous dimensions of risk, via use of a multivariable risk index, which increase power and efficiency [3, 20]. However, despite known benefits to using multivariate risk indices, the frequency of use is unknown.

This study utilizes a large sample of RCTs published in high-impact journals to determine (1) if the use of subgroup analyses and multivariable risk indices has increased, (2) whether statistical methodology has improved over time, and (3) which study characteristics predict subgroup analysis.

## Methods

### Overview

We randomly selected a sample of RCTs published during three time periods in each of five high-impact general medical journals. The search strategy and abstraction forms were developed as part of a previously published study [16]. This study was deemed exempt from the human subject research requirements by the University of Pennsylvania. This study was funded by a grant from the National Pharmaceutical Council.

### Data sources and search

Using a highly sensitive search strategy [21], we searched PubMed for RCTs published in the *Annals of Internal Medicine*, *British Medical Journal* (*BMJ*), *Journal of the American Medical Association* (*JAMA*), *Lancet*, *and* the *New England Journal of Medicine* during the years 2007, 2010, 2013, and the first quarter of 2014. These five journals were selected due to their broad coverage of medical content and substantial impact on medical research and policy [22]. RCTs published during the first quarter of 2014 were included to provide the most recent sample possible and are combined with the 2013 RCT cohort.

Our search yielded 2806 articles. These articles were then randomized into ten batches of approximately 280 articles, stratified by journal and year. Batches (*n* = 4) were randomly selected for assessment of inclusion criteria and full abstraction until we achieved a final sample size of a minimum of 400 included trials.

### Study inclusion

Trials were eligible for inclusion in our sample if they met the following criteria: (1) reported on a human population, (2) reported on a parallel or crossover (including n-of-1) randomized controlled trial, and (3) used randomization at the individual patient level or time within patient (for crossover trials). Nonexperimental designs were excluded, as were cluster-randomized trials, because they often report group-level effects.

### Data abstraction

All studies were independently abstracted by two trained abstracters. Any disagreements were adjudicated by a senior researcher. We used a standard protocol, forms, and electronic database [23] that collected the following information: first author’s last name, journal of publication, year of publication, whether one of the trial authors had formal training in biostatistics (defined as an author holding a terminal degree in statistics, biostatistics, or a related field), medical condition under study, first author’s region (North America, Europe, or other), funding source (any industry funding or no industry funding), the statistical significance of the primary outcome, study design (parallel or crossover), sample size, number of randomized arms, and number of participants randomized to each arm.

Outcomes included the following: (1) any exploration of treatment effect heterogeneity (“subgroup analysis”); (2) among those trials that explored subgroups, use of appropriate statistical methodology (“appropriate methods”); and (3) use of a multivariable risk index to explore subgroups. Subgroup analysis was defined as any reporting of subgroup-specific treatment effects. Subgroup-specific treatment effects included the use of an interaction term in a multivariable model, reporting stratified analyses, or reporting of a single subgroup-specific effect (for example, the treatment effect in women in a study that included both men and women). Appropriate methods for subgroup exploration included applying a test for interaction between the treatment assignment and one or more covariates, or a statistical test of differences in treatment effects *across* subgroups [6, 7]. Solely reporting subgroup-specific effects without a statistical test for heterogeneity was not considered appropriate methodology.

Among the studies reporting subgroup analysis using appropriate methods, we collected a list of the variables examined in the subgroup analysis, including the use of a multivariable risk index. A multivariable risk index is a single variable (usually generated through a multiple logistic regression approach) that captures more than one dimension of risk and allows for risk-based stratification of multiple dimensions [3]; one example is the APACHE score, a severity of disease classification score for critically ill patients. Variables were categorized into the following categories: anthropomorphics, center or site, comorbidities at baseline, demographics, diet and physical functioning, disease severity, history (such as a prior procedure and prior medication exposure), medication at baseline, measures of time (such as season or year), and multivariable risk index.

In order to plot the prevalence of reporting subgroup analysis using appropriate methods over time, we supplemented the current study’s data with those from our prior study [16]. Inclusion of these prior data allows for visualization of six time points over approximately 20 years (1994–2013). The assessment and definition of the subgroup analysis and appropriate methodology was identical to this current study, thereby allowing for combination and direct comparison.

Finally, to assess whether subgroup analysis was reported in a secondary publication, we conducted a forward-citation search of articles that did not report subgroup analysis. Articles that cited these trials were examined to determine if they (1) reported on the same trial participants as the article included in our primary sample; (2) reported subgroup analysis; and (3) if so, used appropriate methods to do so.

### Statistical analysis

Data are summarized as number (percent) or median (range) for discrete and continuous variables, respectively. Bivariable relationships are assessed using chi-square tests. A test for trend is used for the publication year and quintile of sample size. Fisher’s exact test is used when the sample size is small. *P* values of 0.05 or less were considered statistically significant.

Logistic regression analysis was used to examine (1) predictors of subgroup exploration and (2) predictors of using correct methodology for subgroup exploration. Potential predictors include the publication year (entered as a continuous variable), biostatistician as a coauthor, medical condition under study, first author’s region, funding source, the statistical significance of the trial’s primary outcome (defined as significant vs. not significant), and sample size (entered as continuous quintiles). The journal of publication was included in all analyses to control for unmeasured differences across journals. The overall significance of predictors was measured using the Wald test. Predicted probabilities of reporting any subgroup analysis or using appropriate methodology to report subgroup analysis was calculated using the marginal standardization method. This method reflects a weighted average over the distribution of confounders and allows inference to the total population.

Post hoc, we conducted an exploratory analysis examining the potential interaction between funding source and the overall significance of the trial’s primary outcome because a prior study [13] indicated that the overall significance of the trial’s primary outcome moderated the effect between funding and subgroup exploration.

Finally, we conducted an analysis restricted to studies reporting an overall sample size of at least 250 participants, with at least 100 randomized per arm, to determine if similar trial characteristics were associated with subgroup reporting among trials with greater potential for such reporting based on sample size and distribution.

## Results

**Fig. 1:**
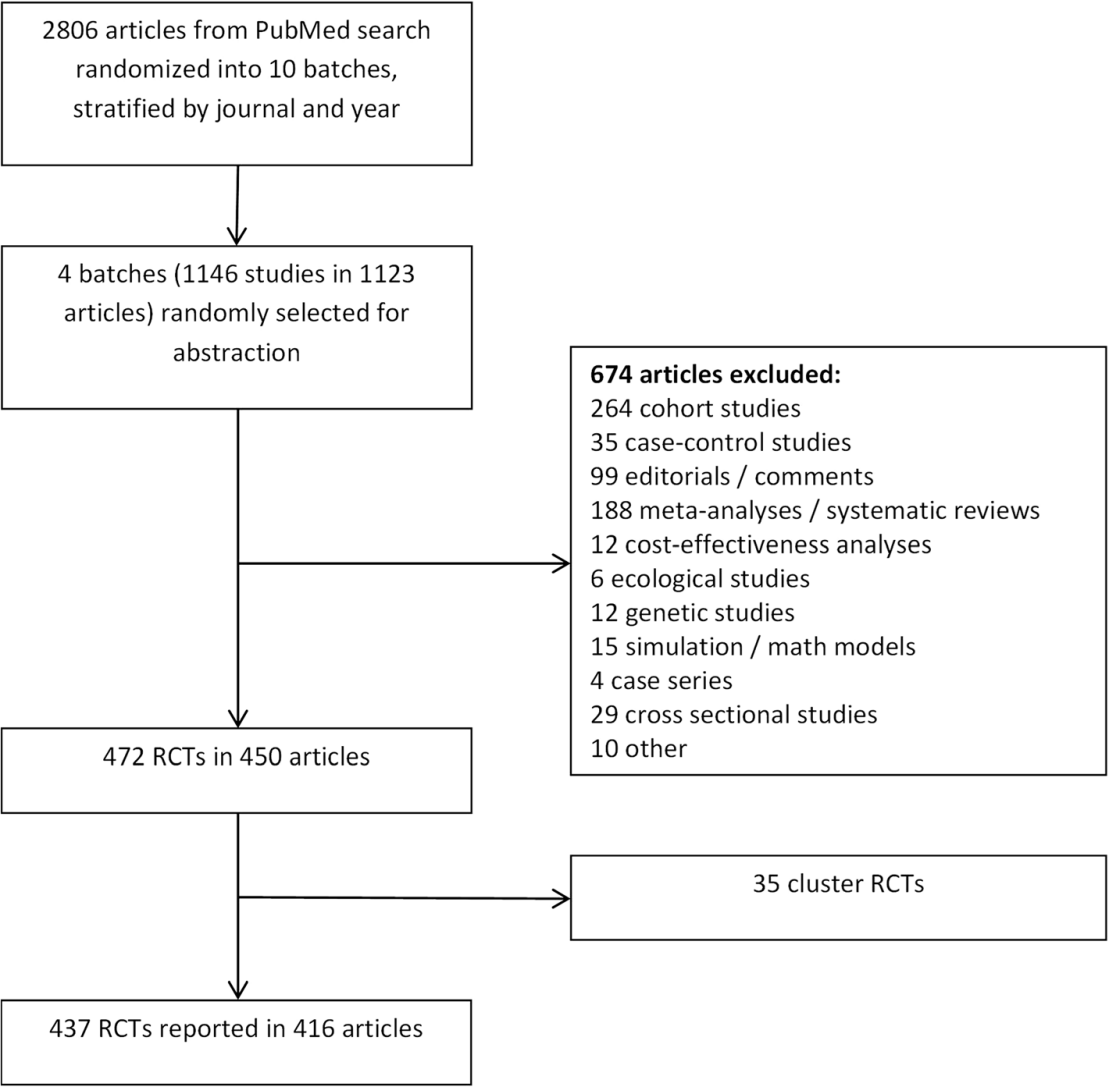
Study search and selection flow diagram

Four batches of papers comprising 1123 articles (representing 1146 studies) were randomly selected for screening. A total of 674 articles were excluded for having trial designs that were not randomized controlled trials, and an additional 35 were excluded for being cluster-randomized trials (Fig. 1). The most common study designs that were excluded were cohort studies (264 (39 %)), reviews (including meta-analysis and systematic reviews) (188 (29 %)), and editorials/commentaries/news articles (99 (15 %)). The 437 included trials (38 % of the initial 1146 studies) were contained in 416 articles: 19 articles reported on more than one RCT, 17 reported on two RCTs, and two articles reported on three RCTs. Articles could report on an included and an excluded study.

Among the included RCTs, the most common medical conditions under study were cardiovascular (23 %), cancer (19 %), and psychiatry/neurology (14 %) (Table 1). The majority of first authors were from either North America (42 %) or Europe (43 %), more than half (57 %) of the RCTs did not receive any funding from industry, and 58 % included a biostatistician as a named coauthor. The vast majority of the trials (97 %) had a parallel group trial design, 64 % of the trials reported a statistically significant primary outcome analysis, and sample size ranged from 7 to 170,432 with a median of 506 participants. More than half (62 %; 270/437) of the included RCTs reported some subgroup analysis. Of the 270 RCTs reporting some subgroup analysis, 185 (69 %) used correct methodology.

**Table 1:** Articles and randomized controlled trials (RCTs) included in the final sample

Characteristic	Articles represented *N* = 416	RCTs included *N* = 437
Journal of publication		
*Annals*	36 (9)	37 (8)
*British Medical Journal*	47 (11)	48 (11)
*Journal of the American Medical Association*	72 (17)	76 (17)
*Lancet*	115 (28)	123 (28)
*New England Journal of Medicine*	146 (35)	153 (35)
Year of publication		
2007	113 (27)	119 (27)
2010	140 (34)	144 (33)
2013–2014	163 (39)	174 (40)
Biostatistician as coauthor	239 (57)	254 (58)
Medical condition under study	--	
Cardiovascular		101 (23)
Infectious disease		82 (19)
Cancer		59 (14)
Psychiatry/neurology		40 (9)
Autoimmune, including diabetes		37 (8)
Pulmonary/critical care		29 (7)
Obstetrics/gynecological		21 (5)
Other chronic disease		41 (9)
Other, uncategorized		27 (6)
First author’s region	--	
North America		185 (42)
Europe		188 (43)
Other		64 (15)
Funding	--	
Industry funding		188 (43)
No industry funding		249 (57)
Significance of the primary outcome^a^	--	
Not significant		153 (36)
Significant		277 (64)
Study design	--	
Parallel group		425 (97)
Crossover		12 (3)
Analysis reported	--	
Subgroup analysis with appropriate methods		185 (42)
Subgroup analysis without appropriate methods		85 (19)
No subgroup analysis		167 (38)
Sample size	--	506 (7–170,432)

*n* (%) or median (range)

^a^ *n* = 7 trials were excluded for not reporting a statistical test for the primary outcome

The results of bivariable analyses examining the relationships between study characteristics and (1) subgroup analysis or (2) using appropriate methods for subgroup analysis are reported in Table 2. Briefly, the journal of publication (*p* = 0.01), medical condition under study (*p* = 0.003), funding (*p* < 0.001), significance of the primary outcome (*p* = 0.013), study design (*p* = 0.002), and sample size (*p* < 0.001) were all significantly associated with reporting any subgroup analysis. Only the year of publication (*p* = 0.046), medical condition under study (*p* < 0.001), funding source (*p* = 0.005), significance of primary outcome (*p* = 0.003), and sample size (*p* = 0.01) were associated with using appropriate methods to conduct subgroup analysis.

**Table 2:** Bivariable associations between trial characteristics and reporting of any exploration of subgroup analysis and reporting of subgroup analysis using appropriate methods

Characteristic	Number	Reports subgroup analysis, *n* (%) *N* = 270	Uses appropriate methods, *n* (%) *N* = 185
Journal of publication		*p* = 0.01	*p* = 0.22
*Annals*	37	21 (57)	18 (86)
*British Medical Journal*	48	21 (44)	12 (57)
*Journal of the American Medical Association*	76	44 (58)	33 (75)
*Lancet*	123	76 (62)	52 (68)
*New England Journal of Medicine*	153	108 (71)	70 (65)
Year of publication		*p* = 0.27	*p* = 0.046
2007	119	74 (62)	57 (77)
2010	144	79 (55)	54 (68)
2013–2014	174	117 (67)	74 (63)
Biostatistician as coauthor		*p* = 0.07	*p* = 0.38
No biostatistician as coauthor	183	104 (57)	68 (65)
Biostatistician as coauthor	254	166 (65)	117 (70)
Medical condition under study		*p* = 0.003	*p* < 0.001
Cardiovascular	101	73 (72)	63 (86)
Infectious disease	82	52 (63)	25 (48)
Cancer	59	45 (76)	24 (53)
Psychiatry/neurology	40	20 (50)	11 (55)
Autoimmune, including diabetes	37	22 (59)	15 (68)
Pulmonary/critical care	29	14 (48)	11 (79)
Obstetrics/gynecological	21	10 (48)	8 (80)
Other chronic disease	41	24 (59)	19 (79)
Other, uncategorized	27	10 (37)	9 (90)
First author’s region		*p* = 0.14	*p* = 0.19
North America	185	123 (66)	84 (68)
Europe	188	113 (60)	82 (73)
Other	64	34 (53)	19 (56)
Funding		*p* < 0.001	*p* = 0.005
Industry funding	188	141 (75)	86 (61)
No industry funding	249	129 (52)	99 (77)
Significance of the primary outcome^a^		*p* = 0.013	*p* = 0.003
Not significant	153	106 (69)	85 (80)
Significant	277	158 (57)	100 (63)
Study design		*p* = 0.002	*p* = 0.53)
Parallel	425	268 (63)	18 (69)
Crossover	12	2 (17)	1 (50)
Sample size		*p* < 0.001	*p* = 0.01
Quintile 1 (median = 69)	88	29 (33)	13 (45)
Quintile 2 (median = 234)	87	44 (51)	30 (68)
Quintile 3 (median = 507)	88	58 (66)	39 (67)
Quintile 4 (median = 1080)	87	65 (75)	48 (74)
Quintile 5 (median = 5455)	87	74 (85)	55 (74)

For articles that report on the appropriate use of methods for subgroup analysis, the denominator used is the number reporting any subgroup analysis

Chi-square tests were used for categorical variables. In the case of small cells, we used Fishers exact test. A test for trend was used for the year and sample size

^a^ *n* = 7 trials were excluded for not reporting a statistical test for the primary outcome

Among the trials reporting subgroup analysis using appropriate methods, the most common variables examined were disease severity (reported in 69 % of studies), demographics (reported in 67 % of trials), baseline comorbidities (31 %), and baseline medication (28 %) (Additional file 1: Table S1). Of the studies that reported subgroup analysis on demographics, age (87 %) and sex (73 %) was the most common. Only 33 studies (18 %) examined subgroups using a multivariable risk index.

**Fig. 2:**
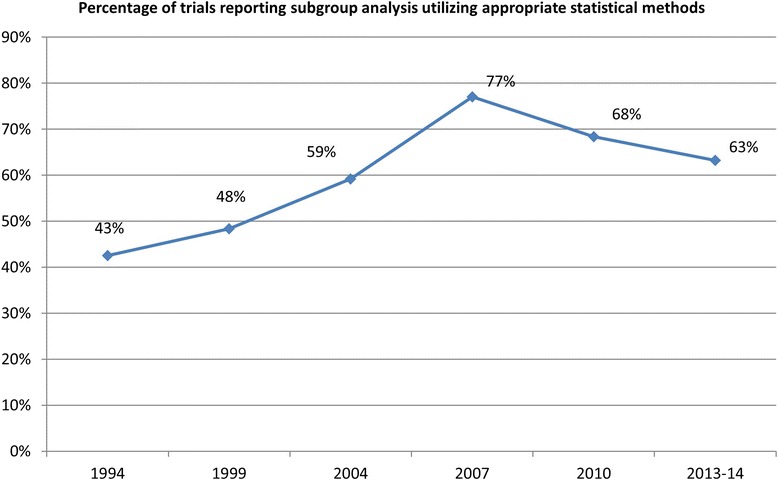
Percentage of trials reporting subgroup analysis utilizing appropriate statistical methods

Figure 2 illustrates the percentage of trials reporting subgroup analysis using appropriate statistical methods over time. Year (2007, 2010, and 2013) showed a significant decrease in the bivariable and adjusted analysis, and additionally, the inclusion of data for 1994, 1999, and 2004 [16] show that peak appropriate reporting occurred in 2007 and decreased thereafter. Reporting percentage using appropriate methods was less than 50 % for the earlier time points (43 % in 1994 and 48 % in 1999), increased to 77 % in 2007, and then decreased to 63 % in 2013.

In a multivariable logistic regression, we found that trials receiving industry funding were more likely to report any subgroup analysis (OR 1.94 (95 % CI 1.17, 3.21)) as were studies with a greater sample size (1.98 (1.64, 2.40) per sample size quintile), but studies with a significant primary outcome were less likely to report subgroup analysis (0.55 (0.33, 0.92) (Table 3). In an analysis restricted to trials reporting subgroup analysis, year of publication, medical condition under study, and funding source were all significant predictors of using appropriate methods. More recent studies were less likely to report subgroups using appropriate methods (0.88 (0.76, 1.00)), as illustrated in Fig. 2. In contrast to the analysis predicting subgroup analysis, industry funding was associated with reduced odds of using appropriate methods (0.39 (0.120, 0.77)). The predicted probability for reporting subgroup analysis was 69 % (95 % CI 62, 75 %) for trials that received industry funding and 56 % (51, 62 %) for studies that did not receive industry funding, but the probability of using appropriate methods to do so was 62 % (54 %, 69 %) for industry-funded studies and 78 % (71, 85 %) for nonindustry funded studies (Table 4).

**Table 3:** Adjusted odds ratios for reporting any exploration of subgroup analysis and for reporting subgroup analysis using appropriate methods

Condition	Predict any subgroup analysis OR (95 % CI)	Predict subgroup analysis using appropriate methods OR (95 % CI)
Journal of publication	*p* = 0.60	*p* = 0.15
*British Medical Journal*	1.00	1.00
*Annals*	1.28 (0.43, 3.82)	7.12 (0.92, 55.31)
*Journal of the American Medical Association*	1.34 (0.53, 3.35)	2.40 (0.63, 8.37)
*Lancet*	1.35 (0.60, 3.03)	3.58 (1.18, 10.88)
*New England Journal of Medicine*	1.90 (0.80, 4.48)	2.28 (0.73, 7.11)
Year of publication	*p* = 0.39	*p* = 0.05
Year	1.04 (0.95, 1.15)	0.88 (0.76, 1.00)
Biostatistician as coauthor	*p* = 0.90	*p* = 0.11
No biostatistician as coauthor	1.00	1.00
Biostatistician as coauthor	1.03 (0.63, 1.68)	1.71 (0.89, 3.30)
Medical condition under study	*p* = 0.10	*p* = 0.02
Obstetrics/gynecological	1.00	1.00
Cardiovascular	2.47 (0.83, 7.35)	2.30 (0.38, 14.02)
Infectious disease	2.03 (0.61, 6.77)	0.51 (0.08, 3.07)
Cancer	3.60 (1.09, 11.84)	0.46 (0.08, 2.67)
Psychiatry/neurology	3.03 (0.87, 10.63)	0.47 (0.07, 3.08)
Autoimmune, including DM	3.53 (1.03, 12.16)	0.71 (0.10, 4.98)
Pulmonary/critical care	1.37 (0.36, 5.22)	1.88 (0.22, 15.85)
Other chronic disease	4.80 (1.41, 16.35)	1.27 (0.18, 8.85)
Other, uncategorized	1.29 (0.37, 4.45)	3.07 (0.22, 43.87)
First author’s region	*p* = 0.65	*p* = 0.47
Other	1.00	1.00
North America	1.48 (0.65, 3.40)	1.51 (0.56, 4.05)
Europe	1.38 (0.63, 2.98)	1.78 (0.71, 4.44)
Funding	*p* = 0.01	*p* = 0.007
No industry funding	1.00	1.00
Industry funding	1.94 (1.17, 3.21)	0.39 (0.20, 0.77)
Significance of the primary outcome	*p* = 0.022	*p* = 0.19
Not significant	1.00	1.00
Significant	0.55 (0.33, 0.92)	0.64 (0.33, 1.24)
Sample size	*p* < 0.001	*p* = 0.18
Quintiles	1.98 (1.64, 2.40)	1.21 (0.91, 1.61)

Model included all variables and robust error terms; *DM* Diabetes Mellitus

**Table 4:** Predicted probabilities for any exploration of subgroup analysis and for reporting subgroup analysis using appropriate methods

Condition	Predicted probability (any subgroup analysis)	Predicted probability (subgroup analysis using appropriate methods)
Journal of publication		
*British Medical Journal*	0.55 (0.41, 0.69)	0.52 (0.33, 0.71)
*Annals*	0.57 (0.42, 0.73)	0.84 (0.63, 1.05)
*Journal of the American Medical Association*	0.60 (0.49, 0.70)	0.70 (0.55, 0.86)
*Lancet*	0.60 (0.53, 0.68)	0.76 (0.67, 0.85)
*New England Journal of Medicine*	0.66 (0.59, 0.73)	0.67 (0.59, 0.75)
Year of publication		
2007	0.61 (0.54, 0.69)	0.77 (0.68, 0.87)
2010	0.57 (0.50, 0.64)	0.71 (0.61, 0.80)
2013–2014	0.66 (0.59, 0.72)	0.65 (0.57, 0.73)
Biostatistician as coauthor		
No biostatistician as coauthor	0.61 (0.55, 0.68)	0.65 (0.56, 0.74)
Biostatistician as coauthor	0.61 (0.56, 0.70)	0.73 (0.67, 0.80)
Medical condition under study		
Obstetrics/gynecological	0.43 (0.25, 0.61)	0.71 (0.42, 1.00)
Cardiovascular	0.61 (0.52, 0.70)	0.85 (0.75, 0.95)
Infectious disease	0.57 (0.46, 0.69)	0.58 (0.44, 0.73)
Cancer	0.68 (0.57, 0.78)	0.56 (0.41, 0.70)
Psychiatry/neurology	0.64 (0.51, 0.77)	0.54 (0.32, 0.76)
Autoimmune, including DM	0.69 (0.57, 0.82)	0.67 (0.46, 0.87)
Pulmonary/critical care	0.50 (0.32, 0.68)	0.83 (0.64, 1.00)
Other chronic disease	0.75 (0.65, 0.85)	0.79 (0.64, 0.95)
Other, uncategorized	0.48 (0.33, 0.64)	0.89 (0.68, 1.00)
First author’s region		
Other	0.56 (0.44, 0.69)	0.63 (0.48, 0.78)
North America	0.63 (0.57, 0.69)	0.70 (0.61, 0.79)
Europe	0.62 (0.55, 0.68)	0.73 (0.65, 0.81)
Funding		
No industry funding	0.56 (0.51, 0.62)	0.78 (0.71, 0.85)
Industry funding	0.69 (0.62, 0.75)	0.62 (0.54, 0.69)
Significance of the primary outcome		
Not significant	0.68 (0.62, 0.75)	0.75 (0.66, 0.84)
Significant	0.58 (0.52, 0.63)	0.67 (0.61, 0.74)
Sample size		
Quintile 1	0.28 (0.17, 0.38)	0.51 (0.27, 0.75)
Quintile 2	0.54 (0.44, 0.63)	0.70 (0.56, 0.84)
Quintile 3	0.67 (0.57, 0.77)	0.70 (0.58, 0.81)
Quintile 4	0.72 (0.63, 0.82)	0.75 (0.66, 0.84)
Quintile 5	0.85 (0.78, 0.93)	0.72 (0.62, 0.82)

Predicted probabilities were calculated using the marginal standardization method; *DM* Diabetes Mellitus

The use of a multivariable risk index for subgroup analysis using appropriate methods is reported in Additional file 1: Table S2. None of the baseline study characteristics were significantly associated with use of a risk index in bivariable analyses, although the sample size is nearly significant (*p* = 0.06), with studies with larger sample sizes more likely to use a risk index when compared to studies with smaller sample sizes.

A sensitivity analysis restricting to trials with a sample size of at least 250 participants and 100 participants per randomized arm (Additional file 1: Table S3) showed similar results to main bivariable analyses. The medical condition under study (*p* = 0.004), funding source (*p* < 0.001), significance of the primary outcome (*p* = 0.015), and sample size (*p* < 0.001) were all significantly associated with the reporting of subgroup analysis, while medical condition under study (*p* < 0.001), funding source (*p* = 0.01), and significance of the primary outcome (*p* = 0.006) were significantly associated with using correct methods for reporting subgroup analysis (Additional file 1: Table S4, appendix). As in the main analysis, industry funding was significantly positively associated with subgroup analysis (85 vs. 63 %) but negatively associated with reporting subgroups using appropriate methods (65 vs. 81 %).

No significant interaction was observed between the funding source and the significance of the primary outcome for reporting any subgroup analysis (*p* = 0.15 for interaction) or for using appropriate methods (*p* = 0.59 for interaction).

In a forward citation search that included 167 RCTs not reporting any subgroup analysis, we found that 35 RCTs reported subgroup analysis in a future publication. Of these, only seven (20 %) used appropriate methodology. In total, of the 437 RCTs in our sample, 305 (70 %) reported on some subgroup analysis, including a later publication, and 192 (63 %) used appropriate methods to do so.

## Discussion

In this large random sample of RCTs in high-impact general medical journals, we found that roughly two thirds of RCTs reported subgroup analysis, and roughly two thirds of those trials used appropriate methodology to conduct such analyses. Furthermore, we found that the percentage of trials using appropriate methodology has, if anything, decreased during the past decade. Furthermore, we showed a relationship with industry funding and subgroup exploration and use of appropriate methods such that industry funding increased the odds of subgroup analysis but decreased the odds of doing so using appropriate methods. Finally, we showed that use of risk indices to explore subgroup is rare. Given that higher-impact journals tend to more frequently report subgroup analyses [13], our estimates likely represent upper bounds for these important practices.

In addition to building on previous work in this area [16], this study explores a different dimension of subgroup analysis, namely utilizing correct methodology among those studies that choose to explore subgroups. Using similar inclusion and coding criteria as the prior study allows us to combine data from the earlier study to examine subgroup reporting over time, including the use of appropriate methodology. While the prior manuscript showed an increase in appropriate methodological use over time, more recent data showed a decrease. Whether this is an artifact of the data, possibly due to chance variation, and smaller numbers within each year, or whether it is a true decrease, is unknown. Given the importance of using appropriate methods and that these methods are well documented in guidance documents [9], monitoring this trend into the future is important to ensure that methodology standards are not slipping.

This is the first study to report the prevalence of subgroup exploration while acknowledging that such analyses may be reported in a secondary manuscript. Our forward search found that an additional 21 % of RCTs reported subgroup analysis in a later publication, for a combined estimate of 70 %. Prior estimates of subgroup reporting have ranged from 40–65 % [11–13, 16–19, 24]; our combined estimate suggests that those prior figures may be underestimates, but that even with this fuller picture of subgroup reporting, roughly one-third of trials originally published in high-impact journals never report subgroup effects.

In addition, our analysis found that 69 % of RCTs reporting subgroup analysis used appropriate methods, but our expanded search into secondary publications found that only 20 % (7 out of 35) of RCTs that reported subgroup analysis in secondary publications used appropriate methods. Whether this is a true effect or due to selection bias and small numbers is unknown, but the potential for substandard reporting in secondary publications (especially those in lower-tier journals) warrants further research.

Other studies [13–15] have also examined the role of industry funding in subgroup exploration and analysis. Our results corroborate prior claims that industry-sponsored RCTs report subgroup analysis more frequently [13, 15]. While other studies did not examine the appropriateness of methods across funding categories, Sun et al. [13] showed that the significance of overall trial results affected whether or not subgroup analyses were reported, with industry-funded RCTs more likely to report subgroup effects if the overall treatment effect was null. We did not find the same interaction in our study but did find that a significant primary outcome was associated with less frequent subgroup exploration overall. The DISCO group [15] examined study protocols and found that industry-sponsored trials planned more subgroup analyses than nonindustry trials. Although Sun et al. [14] showed no relationship between industry and the claiming of a subgroup effect, this is a slightly separate question from the likelihood to explore subgroups and the methods used to do so. Industry funded trials may be more likely to examine subgroups due to better funding or to more appropriately target treatments in specific groups of patients. Our finding that industry-funded trials are less likely to use appropriate statistical methods to do so could be explained by a few possibilities. First, industry funded trials may use appropriate methods, but may not report the methods in sufficient detail to be categorized as “appropriate” in this study. Second, these trials may report appropriate methodology, but such reporting is limited to a protocol manuscript or another separate publication. Finally, industry sponsored trials may truly be less likely to use appropriate methods to conduct subgroup analyses. For the first two cases, strict adherence to reporting guidelines will likely equalize any differences currently observed across funding groups. However, if industry-sponsored trials are truly less likely to use appropriate methods to conduct subgroup analyses, these trials may require additional scrutiny to ensure adherence to appropriate analytic techniques. If these findings are confirmed in future studies, then future research involving examining a full picture of publications related to a trial as well as qualitative interviews and surveys of the authors of a sample of the industry and non-industry sponsored trials may help determine whether this is a reporting issue or a methodology one.

The importance of risk indices for identifying differences across subgroups [25–27] while reducing the likelihood of spurious effects due to multiple comparisons is well documented [8, 27]. However, we found that less than 20 % of the RCTs using appropriate methodology to examine subgroups used a risk index, and that risk indices only constituted 3 % of all variables examined for subgroup analysis. One reason for this may be that validated risk indices are not available for use in all conditions. However, research has shown that the number of available multivariable risk indices is increasing every year, at least in cardiology [28], and a recent article has highlighted the benefits of such approaches in the field of critical care [29]. A simulation study [30] showed that it is possible to develop unbiased internal models to explore subgroups across dimensions of risk, which holds promise for future baseline risk stratification in areas where there are no existing risk indices. Given that risk indices increase power [3] and are better able to estimate the benefit (or harm) of an intervention across groups of patients [2], increased research into the development and use of risk indices remains important.

Our results should be interpreted in light of some limitations. First, our random sample only included five general medical journals, and the inclusion of other journals would have likely yielded less favorable results. Second, possibly, the trials did conduct subgroup analyses using appropriate methods and statistical tests but did not report them as such. However, to impact clinical care, subgroups must be publicly reported. Furthermore, the DISCO group [15] found that less than one third of the RCT protocols included planned subgroup analyses, indicating that many analyses that did occur in our sample (and others) were likely unplanned. Preplanning analyses may result in the use of more appropriate techniques.

## Conclusions

Our findings suggest considerable opportunity for improvement in the conduct and reporting of analyses of how treatments differentially impact patient subgroups, even among trials published in the highest-impact journals. More rigorous reporting standards for subgroup analysis, including the use of an iterative process of exploratory followed by confirmatory analyses and encouragement of the use of risk indices are needed. To maximize the return on investment in RCTs, research sponsors and journal editors should develop policies that encourage subgroup exploration using appropriate methodology. Suboptimal reporting of subgroup effects may impact optimal physician-patient decision-making.

## Abbreviations

APACHE, Acute Physiology and Chronic Health Evaluation; BMJ, British Medical Journal; CONSORT, Consolidated Standards of Reporting Trials; HTE, heterogeneity of treatment effects; JAMA, Journal of the American Medical Association; PCORI, Patient-Centered Outcomes Research Institute; RCT, randomized controlled trial

## Additional file

Additional file 1: Table S1. Variables examined for subgroup analysis. **Table S2** Use of risk inex for subgroup exploration using appropriate methods. **Table S3** RCTs that have a sample size greater than 250 and at least 100 per arm. **Table S4** Bivariables associations of trial characteristics and reporting of any exploration of subgroup analysis and reporting of subgroup analysis using appropriate methods among RCTs that have a sample size greater than 250 and at least 100 per arm. (DOCX 26 kb)
