# Supplementary material for: No improvement in the reporting of clinical trial subgroup effects in high-impact general medical journals
Source: Trials. 2016 Jul 16;17:320. doi: 10.1186/s13063-016-1447-5 (PMC4947338; doi:10.1186/s13063-016-1447-5)
Supplement: Additional file 1: Table S1. — Variables examined for subgroup analysis. Table S2 Use of risk inex for subgroup exploration using appropriate methods. Table S3 RCTs that have a sample size greater than 250 and at least 100 per arm. Table S4 Bivariables associations of trial characteristics and reporting of any exploration of subgroup analysis and reporting of subgroup analysis using appropriate methods among RCTs that have a sample size greater than 250 and at least 100 per arm. (DOCX 26 kb) [file 13063_2016_1447_MOESM1_ESM.docx]

**Additional Table S1: Variables examined for subgroup analysis (n=1042)**

|  | All variables examined  (n=1042) | All trials reporting subgroup analysis using appropriate methods  (n=185) |
| --- | --- | --- |
| *Anthropomorphics* | *39 (4)* | *33 (18)* |
| BMI | 27 (69) | 27 (82) |
| Height | 4 (10) | 4 (12) |
| Weight | 1 (3) | 1 (3) |
| Other | 7 (18) | 6 (18) |
| *Center or site* | *30 (3)* | *28 (15)* |
| *Comorbidities at baseline* | *101 (10)* | *58 (31)* |
| Diabetes | 31 (31) | 30 (52) |
| Cardiovascular disease | 35 (35) | 25 (43) |
| Other comorbidities | 35 (35) | 28 (48) |
| *Demographics* | *258 (25)* | *124 (67)* |
| Age | 108 (42) | 108 (87) |
| Sex | 90 (35) | 90 (73) |
| Race or ethnicity | 31 (12) | 29 (23) |
| Smoking status | 14 (5) | 14 (11) |
| Other | 15 (6) | 9 (7) |
| *Diet and physical functioning* | *9 (1)* | *8 (4)* |
| *Disease severity* | *332 (32)* | *127 (69)* |
| Genetic or inherited factors | 41 (12) | 26 (20) |
| Disease class or stage | 55 (17) | 40 (31) |
| Baseline laboratory value | 126 (38) | 70 (55) |
| Other measure of disease severity | 110 (33) | 67 (53) |
| *History* | *58 (6)* | *40 (22)* |
| Prior procedure or surgery | 7 (12) | 6 (15) |
| Prior disease | 35 (60) | 23 (58) |
| Prior medication | 12 (21) | 11 (28) |
| Other | 4 (7) | 4 (10) |
| *Medication at baseline* | *90 (9)* | *51 (28)* |
| *Measures of time* | *40 (4)* | *31 (17)* |
| Time to randomization / treatment | 22 (55) | 18 (58) |
| Season or year | 6 (15) | 6 (19) |
| Other | 12 (30) | 11 (35) |
| *Multivariable risk index* | *36 (3)* | *33 (18)* |
| *Other* | *49 (5)* | *34 (18)* |

**Additional Table S2: Use of risk index for subgroup exploration using appropriate methods**

| **Condition** | **Reports subgroup analysis using appropriate methods**  **N** | **Uses risk index for**  **subgroup analysis**  **n (%)** |
| --- | --- | --- |
| *Journal of publication* |  | p=0.75 |
| Annals | 18 | 2 (11) |
| BMJ | 12 | 2 (17) |
| JAMA | 33 | 4 (12) |
| Lancet | 52 | 11 (21) |
| NEJM | 70 | 15 (21) |
| *Year of publication* |  | p=0.27 |
| 2007 | 57 | 11 (19) |
| 2010 | 54 | 4 (7) |
| 2013-2014 | 74 | 19 (26) |
| *Biostatistician as co-author* |  | p=0.17 |
| No biostatistician as co-author | 68 | 9 (13) |
| Biostatistician as co-author | 117 | 25 (21) |
| *Medical condition under study* |  | p=0.19 |
| Cardiovascular | 63 | 18 (29) |
| Infectious disease | 25 | 3 (12) |
| Cancer | 24 | 6 (25) |
| Psychiatry/Neurology | 11 | 1 (9) |
| Autoimmune, including DM | 15 | 1 (7) |
| Pulmonary / Critical care | 11 | 2 (18) |
| Obstetrics / gynecological | 8 | 0 (0) |
| Other chronic disease | 19 | 1 (5) |
| Other, uncategorized | 9 | 2 (22) |
| *First author’s region* |  | p=0.49 |
| North America | 84 | 14 (17) |
| Europe | 82 | 18 (22) |
| Other | 19 | 2 (11) |
| *Funding* |  | p=0.40 |
| No industry funding | 99 | 16 (16) |
| Industry funding | 86 | 18 (21) |
| *Significance of the primary outcome* |  | p=0.20 |
| Not significant | 85 | 19 (22) |
| Significant | 100 | 15 (15) |
| *Sample size* |  | p=0.06 |
| Quintile 1 | 13 | 0 (0) |
| Quintile 2 | 30 | 4 (13) |
| Quintile 3 | 39 | 9 (23) |
| Quintile 4 | 48 | 7 (15) |
| Quintile 5 | 55 | 14 (25) |

P-values were calculated using Chi-2 or Fisher’s exact test if cell sizes were small. A test for trend was used for year and sample size.

**Additional Table S3: RCTs that have a sample size greater than 250 and at least 100 per arm**

| **Characteristic** | **RCTs included**  **N=294** |
| --- | --- |
| *Journal of publication* |  |
| Annals | 22 (7) |
| BMJ | 28 (10) |
| JAMA | 41 (14) |
| Lancet | 85 (29) |
| NEJM | 118 (40) |
| *Year of publication* |  |
| 2007 | 81 (28) |
| 2010 | 92 (31) |
| 2013-2014 | 121 (41) |
| *Biostatistician as co-author* |  |
| No biostatistician as co-author | 116 (39) |
| Biostatistician as co-author | 178 (61) |
| *Medical condition under study* |  |
| Cardiovascular | 77 (26) |
| Infectious disease | 66 (22) |
| Cancer | 51 (17) |
| Psychiatry/Neurology | 18 (6) |
| Autoimmune, including diabetes | 16 (5) |
| Pulmonary / Critical care | 19 (6) |
| Obstetrics / gynecological | 20 (7) |
| Other chronic disease | 16 (5) |
| Other, uncategorized | 11 (4) |
| *First author’s region* |  |
| North America | 119 (40) |
| Europe | 128 (44) |
| Other | 47 (16) |
| *Funding* |  |
| Industry funding | 138 (47) |
| No industry funding | 156 (53) |
| *Significance of the primary outcome* |  |
| Not significant | 110 (38) |
| Significant | 183 (62) |
| *Study design* |  |
| Parallel group | 294 (100) |
| Crossover | 0 (0) |
| *Analysis reported* |  |
| Subgroup analysis with appropriate methods | 156 (53) |
| Subgroup analysis without appropriate methods | 60 (20) |
| No subgroup analysis | 78 (27) |
| *Sample size* | 920 (261-170,432) |

n(%) or median(range)

**Additional Table S4: Bivariable associations of trial characteristics and reporting of any exploration of subgroup analysis and reporting of subgroup analysis using appropriate methods among RCTs that have a sample size greater than 250 and at least 100 per arm**

| **Characteristic** | **N** | **Reports subgroup analysis, n (%)**  **N=216** | **Uses appropriate methods, n (%)**  **N=156** |
| --- | --- | --- | --- |
| *Journal of publication* |  | p=0.50 | p=0.22 |
| Annals | 22 (7) | 15 (68) | 14 (93) |
| BMJ | 28 (10) | 18 (64) | 11 (61) |
| JAMA | 41 (14) | 28 (68) | 22 (79) |
| Lancet | 85 (29) | 63 (74) | 46 (73) |
| NEJM | 118 (40) | 92 (78) | 63 (68) |
| *Year of publication* |  | p=0.80 | p=0.41 |
| 2007 | 81 (28) | 59 (73) | 45 (76) |
| 2010 | 92 (31) | 67 (73) | 48 (72) |
| 2013-2014 | 121 (41) | 90 (74) | 63 (70) |
| *Biostatistician as co-author* |  | p=0.83 | p=0.20 |
| No biostatistician as co-author | 116 (39) | 86 (74) | 58 (67) |
| Biostatistician as co-author | 178 (61) | 130 (73) | 98 (75) |
| *Medical condition under study* |  | p=0.004 | p<0.001 |
| Cardiovascular | 77 (26) | 65 (84) | 58 (89) |
| Infectious disease | 66 (22) | 42 (64) | 21 (50) |
| Cancer | 51 (17) | 42 (82) | 24 (57) |
| Psychiatry/Neurology | 18 (6) | 12 (67) | 7 (58) |
| Autoimmune, including diabetes | 16 (5) | 12 (75) | 9 (75) |
| Pulmonary / Critical care | 19 (6) | 10 (53) | 9 (90) |
| Obstetrics / gynecological | 20 (7) | 10 (50) | 8 (80) |
| Other chronic disease | 16 (5) | 14 (88) | 12 (86) |
| Other, uncategorized | 11 (4) | 9 (82) | 8 (89) |
| *First author’s region* |  | p=0.26 | p=0.23 |
| North America | 119 (40) | 90 (76) | 65 (73) |
| Europe | 128 (44) | 96 (75) | 73 (76) |
| Other | 47 (16) | 30 (64) | 18 (60) |
| *Funding* |  | p<0.001 | p=0.01 |
| Industry funding | 138 (47) | 117 (85) | 76 (65) |
| No industry funding | 156 (53) | 99 (63) | 80 (81) |
| *Significance of the primary outcome* |  | p=0.015 | p=0.006 |
| Not significant | 110 (38) | 90 (82) | 74 (82) |
| Significant | 183 (62) | 126 (69) | 82 (65) |
| *Sample size* |  | p<0.001 | p=0.37 |
| Quintile 1 (median=355) | 59 (20) | 34 (58) | 24 (71) |
| Quintile 2 (median=571) | 59 (20) | 41 (69) | 28 (68) |
| Quintile 3 (median=921) | 59 (20) | 44 (75) | 30 (68) |
| Quintile 4 (median=1818) | 59 (20) | 48 (81) | 37 (77) |
| Quintile 5 (median=8322) | 59 (20) | 49 (84) | 37 (76) |

Chi-square tests were used for categorical variables. In the case of small cells, we used Fishers exact test. A test for trend was used for year and sample size.
